# Three cases of resuscitative endovascular balloon occlusion of the aorta (REBOA) in austere pre-hospital environment—technical and methodological aspects

**DOI:** 10.1186/s13017-018-0213-2

**Published:** 2018-11-21

**Authors:** J. C. de Schoutheete, I. Fourneau, F. Waroquier, L. De Cupere, M. O’Connor, K. Van Cleynenbreugel, J. C. Ceccaldi, S. Nijs

**Affiliations:** 1 Burn Unit, Queen Astrid Military Hospital, B-1120 Brussels, Belgium; 2 Department of Trauma Surgery, University Hospitals Leuven, B-3000 Leuven, Belgium; 3 Department of Vascular Surgery, University Hospitals Leuven, B-3000 Leuven, Belgium; 4 175th Surgical Detachment, US Army, Fort Campbell, Kentucky, USA

**Keywords:** Resuscitative endovascular balloon occlusion of the aorta, REBOA, Partial REBOA, Shock, Trauma, Pre-hospital, Austere surgery

## Abstract

**Background:**

The present paper describes three cases where ER-REBOA® was used with partial aorta occlusion (AO), by performing a partial resuscitative endovascular balloon occlusion of the aorta or pREBOA, in an austere pre-hospital military environment.

In addition, because no specific REBOA algorithm for pre-hospital environment exists yet, this paper seeks to fill this gap, proposing a new pragmatic REBOA algorithm.

**Methods:**

Belgian Special Operations Surgical Team applied REBOA in three patients according to a decisional algorithm, based on the MIST acronym used for trauma patients. Only 3 ml, in the first instance, was inflated in the balloon to get AO. The balloon was then progressively deflated, and reperfusion was tracked through changes of end-tidal carbon dioxide (EtCO_2_).

**Results:**

Systolic blood pressure (SBP) before ER-REBOA® placement was not higher than 60 mmHg. However, within the first 5 min after AO, SBP improved in all three cases. Due to the aortic compliance, a self-made pREBOA was progressively achieved while proximal SBP was raising with intravenous fluid infusion. Afterwards, during deflation, a steep inflection point was observed in SBP and EtCO_2_.

**Conclusions:**

ER-REBOA® is suitable for use in an austere pre-hospital environment. The MIST acronym can be helpful to select the patients for which it could be beneficial. REBOA can also be performed with pREBOA in a dynamic approach, inflating only 3 mL in the balloon and using the aortic compliance. Furthermore, while proximal SBP can be convenient to follow the occlusion, EtCO_2_ can be seen as an easy and interesting marker to follow the reperfusion.

## Background

Unlike the Combat Application Tourniquet which is now considered a gold standard in both military and civilian medicine as a proximal control for limb exsanguination in an austere environment or combat environment [1], there is currently no equivalent to control a massive non-compressible truncal bleeding until the patient gets surgical treatment.

The ER-REBOA® catheter [2] has recently been developed with the US department of Defense to fill this void [3]. The rationale is to temporary control the bleeding in the abdomen or pelvis using a resuscitative endovascular balloon occlusion of the aorta (REBOA) technique. This technique consists of placing a flexible catheter into the common femoral artery (CFA) rapidly, and maneuvering it into the aorta without using fluoroscopy and inflating a balloon at its tip. This stops blood flow beyond the balloon and thus arterial bleeding in the distal territory and, at the same time, increases afterload and thus coronary and cerebral perfusion. The balloon can either be placed into zone I or into zone III. Zone I extends from the origin of the left subclavian artery to the celiac artery. The balloon can be inflated there in case of intra-abdominal bleeding, e.g., a liver or splenic injury. Zone II extends from the celiac artery to the lowest renal artery. Zone III extends from the lowest renal artery to the aortic bifurcation [4]. A proximal control of a bleeding caused by a pelvic fracture can be achieved with balloon inflation in this zone.

Because of the ischemia in the distal tissues, this occlusion can only be a temporary maneuver. Total occlusion time is still debated but Irahara et al. [5] and Tsurukiri et al. [6] both described a significantly shorter total occlusion time in the survivor group than in the non-survivor group with 46.2 ± 15.0 vs. 224.1 ± 52.1 min (*p* = .002) and 54 ± 10 vs. 90 ± 27 min, respectively.

Nevertheless, it has recently been shown that REBOA can successfully be applied even in the case of an abdominal venous bleeding in a swine model. REBOA improves hemodynamics and lengthens survival time. Blood loss is similar between groups but the rate of bleeding is markedly decreased with REBOA. REBOA appears effective for central venous injuries and provides a sustained period of stabilization and an extended window for surgical intervention [7].

However, even if the REBOA is used in a variety of clinical settings to successfully elevate central blood pressure in the setting of shock [8–10], the evidence base is weak in trauma, with no demonstrated reduction in hemorrhage-related mortality [11]. Moreover, literature shows a mortality of 65% [12]. The cause of such a high mortality rate, even if not described, can potentially come from multiple organ failure, caused by ischemia-reperfusion injury of more than half the body, in particular in visceral organs, if the balloon is placed in zone I [13, 14].

Therefore, in order to reduce this mortality rate, it has recently been suggested to use a partial aorta occlusion (AO) with partial REBOA (pREBOA) to maintain the proximal systolic blood pressure (SBP) higher than 90 mmHg [15, 16]. Partial REBOA results in maintenance of near-baseline carotid blood flow and central mean arterial pressure, while complete REBOA (cREBOA) generates extreme central mean arterial pressure and prolonged supraphysiologic carotid blood flow, as Russo et al. [17] showed. Moreover, less rebound hypotension after balloon deflation is seen in the pREBOA compared with cREBOA groups. Complete REBOA resulted in higher serum lactate than both pREBOA and controls (*p* < .01). Histology reveals early necrosis and disruption of duodenal mucosa in all cREBOA animals, but none in pREBOA animals [8]. In another paper by Matsumura et al. [18], pREBOA results in a better hemodynamic response than cREBOA (improvement in hemodynamics, 92% vs 70%, *p* = .004; achievement of stability, 78% vs 51%, *p* = .007) and allows for longer occlusion duration (median 58 vs 33 min, *p* = .041).

Nevertheless, if Russo defines pREBOA as a partially inflated balloon until a 50% proximal to distal aortic pressure gradient is achieved (this aortic pressure gradient is measured and maintained continuously by manual adjustment of balloon inflation volume), Matsumura gives another definition (a gradual deflation of the balloon in small steps of 1–3 mL, after confirmation of hemodynamic stabilization, aiming to keep SBP at > 90 mmHg).

However, even if the definition of pREBOA varies between authors, it seems to give better results than a cREBOA. In addition to the better hemodynamic response, a pREBOA allows hemostasis through coagulation factors as well. This cannot be achieved if the flow is stopped due to a total AO.

As patients getting REBOA are intubated and ventilated, end-tidal carbon dioxide (EtCO_2_) can be monitored, as already described in the swine model [19]. EtCO_2_ is indeed the partial pressure of carbon dioxide (CO_2_) in respiratory gases at the completion of an exhaled breath. Normal values vary from 35 to 40 mmHg. These values reflect cardiac output, production of CO_2_ and alveolar function. When cardiac output is reduced, CO_2_ delivery to the lungs decreases and EtCO_2_ decreases as well [20]. Moreover, there is a correlation between EtCO_2_ and lactic acidosis [21]. And there is also a strong positive correlation between total occlusion time with REBOA and lactate concentration (*p* = .02) [6]. In this emergency configuration, when intubated, the alveolar function of the patient is defined by the respiratory machine. If the settings of the respirator are not modified, only changes in cardiac output and production of CO_2_ are influencing the EtCO_2_. However, during balloon deflation, changes in cardiac output are visible on the monitoring following the SBP. So, reperfusion can be tracked through changes of EtCO_2_.

Besides this, from a technical point of view, a 7-French sheath compatible REBOA catheter is commercialized in the USA since 2015, which needs only manual compression as closing technique and no closing device due to the diameter. Before that, only minimum 12-French sheath systems existed, and a surgical closure or the use of a closure device was required. Even if the surgeon still has to be able to deal with potential complications, like ongoing bleeding from the puncture point, false aneurysm, artery dissection, thrombo-embolism, or limb-threatening ischemia, the use of this 7-French sheath facilitates the use of REBOA in less technical environments. Moreover, as shown previously by Stensaeth et al. [22], it is possible to use this REBOA catheter without fluoroscopy. Another specific feature of this catheter, which makes it particularly suitable for use in an austere pre-hospital environment, is its self-guiding property in the aorta with its atraumatic tip, hence avoiding the need for a guide wire. Members of a US Air Force Special Operations Surgical Team (SOST) have recently used the ER-REBOA® in an austere military setting [23]. They used a hand-held ultrasound to diagnose hemoperitoneum performing a focused abdominal sonography for trauma (FAST) examination and to facilitate a 7-French femoral sheath access in the CFA. They obtained good results with no access- or REBOA-related complications and with all of their four patients surviving transport to the next echelon of care in stable condition. Besides that paper, Northern et al. described the use of 20 REBOAs in an austere pre-hospital environment by the US Air Force SOSTs [24]. They concluded that REBOA can be used as a pro-active resuscitation strategy and that the proficiency of non-surgeon physicians in the placement of REBOA is a force multiplier. To our knowledge, these are the only pre-hospital experience with the one of the London’s Air Ambulance, although limited to one blind positioning in zone III only [25].

Taking that into consideration, the present paper describes three cases where ER-REBOA® was used in an austere pre-hospital military environment. A dynamic approach, doing on the one hand a pREBOA by inflating or deflating the balloon to keep the SBP not higher than 90 mmHg as well as using on the other hand the aortic compliance, defined as the ability of a vessel to distend and increase volume with increasing transmural pressure or the tendency of a vessel to resist recoil toward its original dimensions on application of a distending or compressing force, was chosen here.

## Methods

### Belgian SOST

Our SOST is a specialized team (comprising a trauma surgeon, an anesthetist, a scrub nurse, an emergency anesthesia and critical care registered nurse, and a paramedic as well as a special force combat medic) providing triage, damage control resuscitation (DCR), and damage control surgery (DCS) in an austere military environment. The team members are trained to deal with forward surgical care. Patients included in the present case series were treated in a casualty collection point (CCP), close to the front line where triage, DCR, and DCS could be performed, in accordance to the NATO doctrine about forward surgical element [26]. Neither X-ray nor laboratory tests were available. The only available technical investigation was ultrasound. The goal of deploying a SOST is to diminish the evacuation time until surgery and to allow a rapid stabilization of unstable patients, which would otherwise die during evacuation to a conventional medical treatment facility (MTF) [26]. On average, about 90 casualties were treated weekly at the location of the SOST. DCS proved necessary for an average of eight cases a week. Three patients were treated with a REBOA according to the decisional algorithm detailed in the next paragraph.

### Treatment rationale using REBOA

**Fig. 1:**
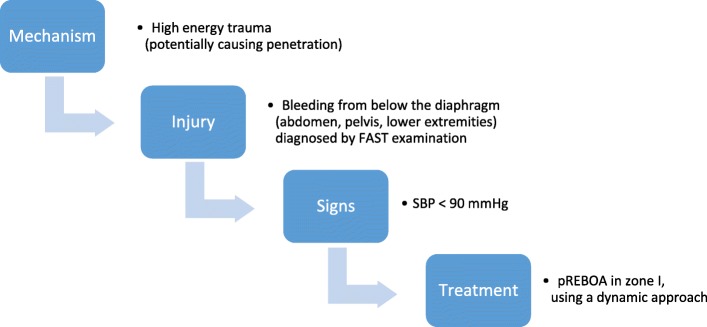
Proposed algorithm for the placement of REBOA catheters in pre-hospital. Due to a lack of diagnostic devices in pre-hospital settings, in-hospital algorithms [45] are impossible to implement. Nevertheless, a pragmatic approach is required to use REBOA in the right patient. Therefore, REBOA catheters can be placed according to this algorithm based on the MIST acronym, widely accepted in pre-hospital care, and quickly useable by any medical provider, regardless of his training level

Due to the lack of diagnostic devices, the high inflow of patients and the limited capacity of the surgical team, a pragmatic approach was required to use REBOA in the right patient. Therefore, ER-REBOA® was placed according to the following algorithm, depicted in Fig. 1 and based on the MIST acronym [27].

- Mechanism of injury: high-energy trauma (potentially causing penetration)
- Injury: following wounds localization and FAST examination in order to exclude any bleeding injury above the diaphragm
- Signs: shock, traditionally defined as SBP < 90 mmHg [28]

Afterwards, for the algorithm-elected patients, a pREBOA strategy following Matsumura et al. [18] was used. That means a gradual deflation of the balloon in small steps of 1–3 mL, after confirmation of hemodynamic stabilization, aiming to keep SBP at > 90 mmHg.

However, the first inflation of the balloon to get AO was smaller in our experience than that described by the ER-REBOA® company [2]. Instead of inflating height milliliters and then checking the effect on SBP, only 3 mL were used here in the first instance to get AO. Doing this, it was possible to use the vasoconstriction/vasodilatation of the aorta to perform a pREBOA. At the beginning, AO was achieved flushing 3 mL in the balloon because the hypovolemic patient was in vasoconstriction. At the same time, intravenous (IV) fluids and blood products were given, increasing the intravascular volume above the balloon. When the proximal SBP had increased, the balloon inflation was insufficient to keep a total AO and permitted some leakage, hence creating a self-made pREBOA through aortic compliance.

The pREBOA, obtained by a combination of small inflation and aortic compliance, brought about a dynamic approach.

### Technique

First of all, it was essential to exclude cardiac tamponade and tension pneumothorax during the primary survey, prior to initiating a REBOA therapy. After that, an ER-REBOA® was inserted in all patients with SBP < 90 mmHg due to high-energy trauma below the diaphragm. Procedure was performed by the surgeon, helped by the scrub nurse. If blind percutaneous arterial access could not be achieved in 30 s, a cut-down procedure at the level of the CFA was performed. The idea was to avoid wasting time with only one technique—the percutaneous one—if it was not immediately successful. A super Arrow-Flex sheath introducer, 7-French (CP-07711), was systematically used. After sheath placement, only external anatomic landmarks [29] were used to position the REBOA catheter. Because the ER-REBOA ® balloon is not positioned at the end of the catheter, it is recommended to place the proximal edge of the balloon at the xiphoid level when measuring for positioning in zone I and to place the proximal edge of the balloon just above the umbilicus when measuring for positioning in zone III [30]. Fixation of the sheath in soft tissues and of the catheter on the skin was performed using Ethilon ® 2/0 sutures. After positioning of the catheter in the aorta, the balloon was inflated with 3 mL to cause total AO but permitting a dynamic approach as described earlier. The right effect of the REBOA balloon was controlled by direct view and/or palpation (depending on the access technique) of the gradual loose of pulsation in the CFA during inflation of the balloon. Later during surgery, the right position of the REBOA balloon was checked by manual palpation of a pulseless aorta just under the diaphragm, the balloon being placed in zone I. Because full deflation of the balloon at once may result in reperfusion injury, causing washout of metabolic byproducts and acidosis, saline was progressively (over 30 min) removed from the balloon as soon as possible during surgery to keep a SBP around 90 mmHg. Effects of balloon deflation on patients were constantly followed by heart rate (HR), SBP, and EtCO_2_. After full deflation, the catheter was removed. Hence, the sheath was withdrawn under visual control, and the artery was closed using one or two Prolene® 5/0 sutures. Indeed, considering the unknown delay to transport the patient to the next MTF, in which circumstances the transport would happen, and if a vascular surgeon would be available at the next local MTF, it was decided not to use a manual compression to close the CFA.

## Results

### REBOA insertion procedure and technique (resumed in Table 1)

Percutaneous access was started in all cases but was only successful for one case under the first 30 s. A cut-down procedure was thus used in the two other cases. Total time needed to get AO varied between 5 min in the fastest case and 9 min in the slowest case. For the first patient, 8 min were needed because it was the first time the team, surgeon excepted, performed the procedure. The slowest ER-REBOA® placement, for the second patient, took 9 min because the surgical instruments were at that time in the autoclave for sterilization, slowing the cut-down procedure. Five minutes were needed for the third patient. No balloon migration was observed after inflation. Due probably to hypovolemia, a balloon inflation of 3 mL was sufficient to get a total AO. The AO was maintained during approximately 30 min with a balloon inflated with 3 ml, and after that, the balloon was gradually deflated over approximately 30 min in all three cases. During the time the balloon was in place, DCR and DCS more particularly were performed.

**Table 1:** REBOA procedures for the patients who required it following the presented algorithm

REBOA procedure	Patient 1	Patient 2	Patient 3
Technique for arterial access	Percutaneous	Cut-down	Cut-down
Volume inflated in balloon to get total AO	3 mL	3 mL	3 mL
Landing zone	Zone I	Zone I	Zone I
Duration of AO with 3 ml	30 min	30 min	34 min
Duration of balloon deflation	35 min	28 min	25 min
Site closure	Stitch	Stitch	Patch
Site complication	None	Thrombosis	Thrombosis

### Clinical evolution

**Fig. 2:**
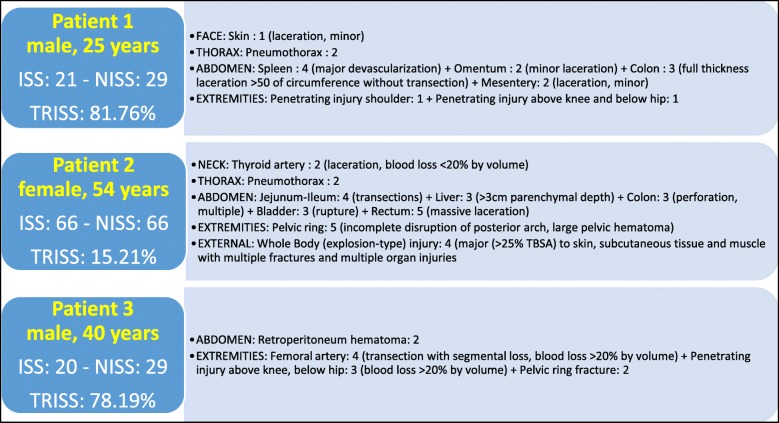
Abbreviated Injury Scale, Injury Severity Score, New Injury Severity Score, and Trauma Injury Severity Score (% of survival) of the three patients. Due to a lack of diagnostic devices, some lesions were missed. The calculated scores are therefore most probably underestimated

All three patients had a high-velocity penetrating trauma, due to an improvised explosive device or gunshot wound. For so far known, they did not have prior history of peripheral vascular disease. The patients were brought to the SOST location directly from the point of injury by non-governmental organization ambulances. Transport time was about 20 min. Cardio-pulmonary resuscitation was not needed during transport. When arriving at the CCP, SBP before ER-REBOA® placement was not measurable and neither was carotid pulse felt in two patients. For the third one, SBP was 60 mmHg. However, within the first 5 min after AO, SBP improved in all cases but it was not higher than 90 mmHg in this first occlusion period. The SBP reached 90 mmHg after a maximum of 34 min after AO. IV fluids and blood products given varied from 3 to 4 L and from 1 to 2 L, respectively. Additionally, vasopressors were also required for two patients to keep SBP high enough, as depicted in Table 2. Surgical hemorrhage control delay varied from 26 to 135 min after AO, depending on the procedures performed: either liver packing and pelvic packing or pelvic packing and contralateral CFA shunting. The abbreviated injury scale (AIS) [31], the injury severity score (ISS) [32], the new ISS (NISS) [33], and the trauma injury severity score (TRISS) [34] were calculated and are detailed in Fig. 2. After sheath removal, two patients developed a thrombosis at the site of sheath placement. One developed it during DCS, when the sheath was in place (patient 3); the other one developed it 1 day later (patient 2). Regarding patient 2, the thrombosis was due to a technical error: the artery had to be closed in quick and dirty circumstances during an evacuation. That limb-threatening, but not life-threatening, complication was managed during the DCS procedure for patient 3 and at the Role 3 MTF for patient 2. Overall, the time at CCP was about 2 h for two patients; the third one stayed about 4 h and 30 min before transfer to the next MTF. Timelines are summarized in Table 3.

**Table 2:** Damage control resuscitation regarding the patients who got a REBOA

Damage control resuscitation	Patient 1	Patient 2	Patient 3
Mechanism of injury	Mounted improvised explosive device	Dismounted improvised explosive device	Gunshot wound
Tranexamic acid	1 g	1 g	1 g
Antibiotics	Cefazolin 2 g Metronidazole 500 mg	Cefazolin 1 g Metronidazole 500 mg	Cefazolin 2 g Metronidazole 500 mg
Vasopressors	None	Epinephrin 1 mg × 2 Phenylephrin 100 μg × 4	Phenylephrin 100 μg × 10
Crystalloids	3 L	3.5 L	4 L
Blood products	2 FWB	4 FWB	2 FWB 3 PRBC
Estimated blood loss	2 L	3 L	4 L

**Table 3:** Timelines at CCP. Times are noted in minutes from the time at admission

Events	Patient 1	Patient 2	Patient 3
Start of AO procedure	71	15	9
Successful AO	79	24	14
Start of surgery	94	26	41
Hemodynamic stability (90 mmHg)	109	24	48
Definitive hemorrhage control	104	50	183
End of surgery	174	105	243
Evacuation	184	115	263

### Evolution in SBP and EtCO_2_ over time

**Fig. 3:**
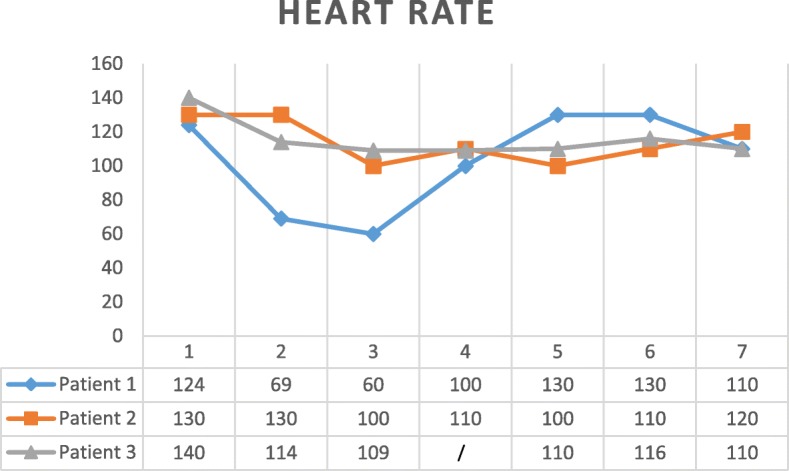
Heart rate during REBOA procedure. 1—at admission, 2—before REBOA, 3—within the first 5 min after AO, 4—after deflation of 1 cc (2 cc were directly deflated for patient 3), 5—after deflation of 2 cc, 6—after full deflation, and 7—at the end of the surgery

Three parameters were followed during the intervention: HR, SBP, and EtCO_2_. Looking at HR evolution, as in Fig. 3, no trend can be observed. However, the two other parameters can be described.

**Fig. 4:**
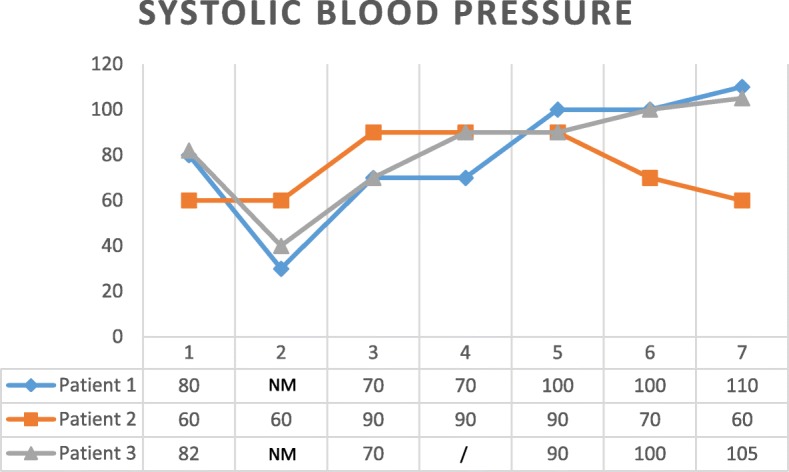
Systolic blood pressure during REBOA procedure. 1—at admission, 2—before REBOA (NM = not measurable for patients 1 and 3), 3—within the first 5 min after AO, 4—after deflation of 1 cc (2 cc were directly deflated for patient 3), 5—after deflation of 2 cc, 6—after full deflation, and 7—at the end of the surgery

- SBP (evolution resumed in Fig. 4)

**Fig. 5:**
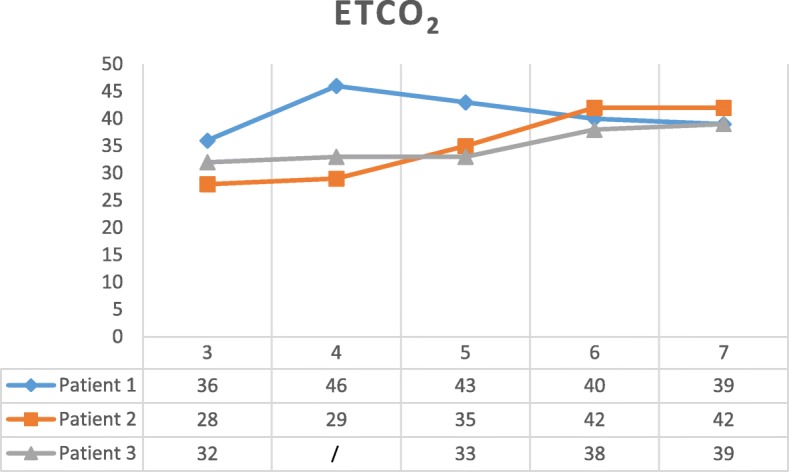
End-tidal carbon dioxide during REBOA procedure. Values at admission and before REBOA were not measured. 3—within the first 5 min after AO, 4—after deflation of 1 cc (2 cc were directly deflated for patient 3), 5—after deflation of 2 cc, 6—after full deflation, 7—at the end of the surgery. A steep inflection point can be observed in 4 for patient 1 and in 6 for the other patients

SBP rose in all cases after balloon inflation. Then, after deflation, SBP further rose in two cases but not in the third one (patient 2). In that one, SBP before starting REBOA was 60 mmHg. Even if SBP rose to 90 mmHg after inflation, it dropped to 60 mmHg afterwards when the balloon was deflated. This happened because surgery had to be interrupted and the catheter had to be removed because the medical evacuation helicopter was coming. Epinephrine 1 mg was then given twice in the next minutes after full deflation to keep SBP as high as possible. Besides the role of ER-REBOA® to maintain an acceptable SBP, other adjuncts were simultaneously used as illustrated in patient 3. Because in that case, SBP was still less than 90 mmHg 30 min after full AO, fluids, blood transfusion, and phenylephrine were given and the deflation process was started. Direct proximal arterial control was achieved 5 min later by clamping the contralateral CFA just above a traumatic transection of that vessel. The balloon was then deflated to 1 mL and not fully deflated because SBP was only 90 mmHg at that time, and blood flow was coming back in the leg where the sheath was placed, causing more hypotension. The idea was to deal with reperfusion gradually. Twenty-five minutes later, full deflation occurred. The consequent dropping SBP was here again compensated giving phenylephrine.

b. EtCO_2_ (evolution resumed in Fig. 5)

EtCO_2_ has not yet been used as parameter to follow reperfusion when the balloon is progressively deflated. Three different curves were observed for the three patients.

For patient 1, EtCO_2_ rose from 36 to 46 after the first deflation. Then, it took 45 min to go back from 46 to 36. The last EtCO_2_ of 39 cannot be correlated to the other ones because the patient was waking up. He was then not on the respiratory machine anymore, and the respiration rate was influencing the EtCO_2_ at that moment.

On another side, for patient 2, it is important to notice that the first EtCO_2_ measured was 28, which is quite low, potentially because the patient was hypothermic and hypovolemic at that moment. A deflation from 3 to 2 mL did not affect EtCO_2_ so much, the other ones did affect, rising from 29 to 35 and then to 42.

However, for patient 3, EtCO_2_ was relatively stable, varying from 32 to 39.

## Discussion

### When to place the REBOA catheter in pre-hospital?

Due to the austere environment, prioritization to allow for the most adequate resource allocation is key. For example, the time between admission at CCP and start of AO procedure was 71 min for patient 1. This is quite a long time, but it is because the SOST was operating on another patient when patient 1 arrived. As already suggested [24], the placement by someone else than the surgeon could have speeded up the treatment of the patient.

Due to a lack of diagnostic devices, usual in-hospital algorithms [4, 35] are impossible to implement. Nevertheless, a pragmatic approach is required to apply REBOA to the right patient. Therefore, ER-REBOA® was placed according to the algorithm depicted in Fig. 1 and based on the MIST acronym [27]. In a pre-hospital setting, the tactical combat casualty care guidelines [1] are required to stop a massive external bleeding at the level of the limbs or REBOA for massive non-compressible truncal bleeding below the diaphragm. Thereby, limited resources like blood can be spared and the patient can reach a conventional MTF where more diagnostic options exist and where further treatment can be given using more resources. Therefore, to be able to act quickly and to make it understandable to all medical care providers, no matter what their training level is, the idea of following the MIST acronym allows for a fast decision-making tool. Here, we used only three packed red blood cells (PRBC) and eight fresh whole blood (FWB) to keep the three patients alive, even with an ISS score between 20 and 66. This highlights the importance of the availability of FWB, as also by the US Air Force observed [24].

### Percutaneous placement or cut-down procedure?

Data from the American Association for the Surgery of Trauma showed that a cut-down procedure is performed in 50% of cases, a percutaneous placement without imaging in 28.3% of cases, and finally, 10.9% of cases are ultrasound guided [36]. This last option was used by the US Air Force SOST [23] to facilitate the femoral sheath access. We used another strategy because ultrasound control was seen as a time-consuming adjunct. If blind percutaneous arterial access could not be achieved in 30 s, then a cut-down procedure at the level of the CFA was performed. This “30-s” window was actually decided to let the surgeon already start with the percutaneous procedure and in parallel let the paramedic find and prepare the instruments for a cut-down procedure, hence avoiding preparation delays. Moreover, cut-down procedure was not seen as a failed procedure because open CFA closure was anyway planned in all cases. So, cut-down procedures were here seen as a helpful way to visualize the loose of pulsation in CFA when inflating the balloon. In addition, the placing of catheters by cut-down procedure was not very time-consuming. The same range of time in a pre-hospital configuration was described by the US team [23] using hand-held ultrasound. Romagnoli et al. [37] performed a resuscitative thoracotomy in a median time of 5.28 min and a REBOA in a median time of 7.9 min, but in a hospital.

### Steep inflection point?

The biggest impact of AO on SBP for patient 2 was noticed when the last milliliter was removed from the balloon, suggesting first an important role of partial AO on the stability of the patient and second a nonlinear correlation between AO and SBP. This observation has actually been described by Davidson et al. [38] in a swine model where a steep inflection point occurs during balloon deflation that results in an abrupt increase in aortic flow and a concomitant decrease in mean arterial pressure. Furthermore, the onset of distal aortic flow was inconsistent across study animals and did not correlate with initial balloon volume or relative deflation volume.

Because HR and EtCO_2_ were recorded here, measurements can be matched with SBP at the same moment. Thus, for patient 1, a deflation from 3 to 2 mL did affect HR and EtCO_2_ much more than the other deflations. This observation suggests that the steep inflection point described for SBP could be followed by HR and EtCO_2_ as well. For patient 2, even if a deflation from 3 to 2 mL did not affect EtCO_2_ so much, the other ones did affect, raising from 29 to 35 and then to 42. The steep inflection point was then seen when deflating the last milliliter from the balloon, as written here above. Here, only a minimal raise of HR was observed at the same moment. For patient 3, even if EtCO_2_ was relatively stable, varying from 32 to 39, a steep inflection point could be seen again when full deflation occurred. Identically, HR was stable but rose a bit at the moment of full deflation as well. However, this was less marked than for the two other patients.

### Manual compression or surgical closure?

When a cut-down procedure is performed to place the sheath, there is no other choice than to surgically close the CFA. However, for the third patient, both options were available. Even if the ER-REBOA® is placed using a 7-French sheath, which allows for manual compression instead of a closing device, the surgical team is confronted to some important questions about the continuum of care in austere circumstances. Who will take care of the patient at a next local MTF? When will the patient arrive at the next local MTF? Do they have ultrasound at the next local MTF? What will happen if the CFA is bleeding during a difficult transport? Will the local ambulance team be able to deal with a bleeding? Will the patient not bleed due to trauma-related coagulopathy?

However, we observed a thrombosis in two of our patients, one before surgical closure and one after. The latter was due to a technical error, and no clear cause was identified for the former. This can perhaps be linked to a mix of a hypercoagulation status due to stress, fresh whole blood transfusion, vasopressors, and tranexamic acid.

But our data, although limited, are in contradiction with other authors who observed no complication related to sheath insertion or removal, including dissection, pseudoaneurysm, retroperitoneal hematoma, leg ischemia, or distal embolism [9, 39].

### To leave the material in place for transport to next MTF?

One patient (patient 2) was transported to a NATO Role 2 MTF. There, the patient was reanimated. During reanimation, the surgeon removed the abdominal temporary closure device and compressed directly with his hand the aorta just under the diaphragm. The patient could recover and was then transported to a Role 3 MTF where further surgery and medical treatment was given until transport to a Role 4 MTF at day 4 post injury. This situation raises some considerations.

It would have been easier to monitor SBP if the sheath was left in place for transport. Moreover, it would have been easier to keep the patient stable with the catheter in place, still allowing some perfusion of distal tissue doing pREBOA. This could potentially have permitted a straight evacuation to the Role 3 MTF without any stop at the Role 2 MTF. This means that the patient has been brought faster to an MTF with a higher level of care. However, personnel need first to be confident with REBOA, being then able to take care of the catheter during transport. Second, if short transport is not possible, the evacuation team needs to be comfortable with flushing the sheath with saline on the 20 min to avoid thrombosis. Third, the transport team needs to be trained for potential massive CFA bleeding if there is any movement on the sheath causing a leak.

### Which training and for whom?

Eastridge et al. [40] showed in a study about battlefield fatalities that 87.3% of all injury mortality occurred in the pre-MTF environment and that 24.3% were deemed potentially survivable. The injury/physiologic focus of potentially survivable acute mortality was largely associated with hemorrhage (90.9%). Furthermore, the site of lethal hemorrhage was truncal (67.3%), followed by junctional (19.2%) and peripheral-extremity (13.5%) hemorrhage. This means that it could be interesting to train pre-MTF personnel (e.g., SOST) to REBOA skills to reduce that potentially survivable acute mortality.

Virtual reality simulation showed good results in learning damage control endovascular procedures to doctors without any REBOA experience [41], reducing procedural task times (the task began when the guide wire entered the femoral arterial catheter and ended with the inflation of the balloon) from more than 4 min the first time to 2 min the sixth time. Besides this simulation, other possibilities exist as well like the endovascular skills for trauma and resuscitative surgery curriculum [42] or the endovascular and hybrid trauma and bleeding management workshop [43]. However, nothing about the skill decay in time has been described yet.

In our experience, REBOA procedure was reduced from 8 min at the beginning to 5 min after two times. Nevertheless, because, in an austere environment with only a few resources, coordination among team members is paramount, we would encourage the whole SOST to follow a simulation course. We saw indeed that the skills of the scrub nurse and paramedic were the time limitation factor in our series.

### Limitations

The dynamic approach, obtained by combination of balloon small inflation and aortic compliance, was based on a physical rationale and not on any measurement. Therefore, measuring this on an animal model in the future would better define this physical rationale.

Another limitation of this report is its retrospective nature and the fact that it describes only a series of three cases.

Additionally, it was not possible to keep long-term data for the patients due to the transfer in local MTF for further treatment.

## Conclusions

### ER-REBOA® in pre-hospital austere environment

Due to his feature, this catheter is particularly suitable for use in an austere pre-hospital environment, as shown by Manley et al. [23] and here. Moreover, as described in Fig. 1, it is efficient to use it following the MIST acronym. This means that when (1) the patient sustained a high-energy trauma, (2) a lower limb external bleeding could not be controlled by manual compression nor a tourniquet or an internal bleeding from the abdomen or pelvis was diagnosed with the hand-held ultrasound, and (3) SBP was below 90 mmHg during the triage and resuscitation. The catheter is then used to buy time to get the patient alive on the operation table. From that moment, DCS can be performed, the source of the bleeding can be located, and the balloon can be gradually deflated. Furthermore, in an austere environment where resources are limited, temporary blocking inflow of blood below the diaphragm improves the surgical performance by facilitating perioperative view as well as reducing the need for blood transfusion. This, thus, saves the limited blood supply, and it reduces trauma coagulopathy. Finally, even a patient with an as low as non-measurable and not felt SBP can still be resuscitated out of a hospital if temporary AO is obtained.

### Dynamic approach using pREBOA

In our opinion, REBOA should not be approached as an “ON-OFF” technique. As shown here, REBOA can also be used with a partial AO or pREBOA in a dynamic approach. With 3-mL inflation in the balloon, enough proximal control of the bleeding can be reached to raise the SBP, using simultaneously other resuscitation ways like IV fluids, blood products, vasopressors, and light anesthesia. Furthermore, while SBP can be used to follow the occlusion, HR but especially EtCO_2_ can both be seen as easy and interesting markers to follow the reperfusion and the steep inflection point.

From our experience, further considerations can arise, and we mention three of them. First, because patients can become hemodynamically unstable at the onset of sedation, would it then be better to already place the sheath using local anesthetic before sedating the patient, perhaps already during the primary survey? Second, because the surgeon is focused on his surgery during DCS, would it not then be better to let the anesthetist inflate and deflate the balloon of the REBOA and follow adequately the inflation time? Third, because pREBOA in a dynamic approach is an adjunct for resuscitation, would it then be helpful to use REBOA during transport between two MTF as suggested by Reva [44]? For such a usage, more people in the whole chain of evacuation, also from the transport team, would definitely need to comprehend this relatively new paradigm and to be confident with the material. However, some initiatives already exist in this sense [41–43].

## Abbreviations

AIS: Abbreviated Injury Scale
AO: Aorta occlusion
CCP: Casualty collection point
CFA: Common femoral artery
CO_2_: Carbon dioxide
cREBOA: Complete resuscitative endovascular balloon occlusion of the aorta
DCR: Damage control r
DCS: Damage control surgery
EtCO_2_: End-tidal carbon dioxide
FAST: Focused abdominal sonography for trauma
FWB: Fresh whole blood
HR: Heart rate
ISS: Injury severity score
IV: Intra-venous
MTF: Medical treatment facility
NISS: New injury severity score
PRBC: Packed red blood cells
pREBOA: Partial resuscitative endovascular balloon occlusion of the aorta
REBOA: Resuscitative endovascular balloon occlusion of the aorta
SBP: Systolic blood pressure
SOST: Special Operations Surgical Team
TRISS: Trauma injury severity score
